# Testing the potential of 50 kHz rat calls as a species-specific rat attractant

**DOI:** 10.1371/journal.pone.0211601

**Published:** 2019-04-08

**Authors:** Nicola B. Davidson, Jane L. Hurst

**Affiliations:** Mammalian Behaviour and Evolution Group, Institute of Integrative Biology, University of Liverpool, Leahurst Campus, Neston, Cheshire, United Kingdom; University of Missouri Columbia, UNITED STATES

## Abstract

The control of mammalian pests relies heavily on the use of pesticides that are often avoided and are not species-specific. These problems are particularly acute for pesticides used to control rats (*Rattus spp*.). The efficacy and targeting of control could be improved by attracting animals to control measures using species-specific cues. One cue that has the potential to attract rats is the 50 kHz calls they emit in positive social situations. Here we test the potential of these rat calls as a species-specific attractant by examining the response of laboratory rats (*Rattus norvegicus*; n = 48) and non-target bank voles (*Myodes glareolus*; n = 16) to 50 kHz calls from either sex in a compartmentalised laboratory arena. Sounds of rat movement and white noise acted as control treatments, with each sound tested against a silent control in the opposite side of the arena. When sound cues were played above an empty bait box, rats were attracted to spend time close to 50 kHz rat calls, climbing on top of boxes, regardless of the sex of subject or caller. When either 50 kHz rat calls or rat movement sounds were played inside an empty bait box, rats of both sexes spent 3–4 fold more time inside boxes and visited more frequently. Rats were not attracted by intermittent white noise. Bank voles were neither attracted to, nor avoided, 50 kHz rat calls played inside empty bait boxes. Our findings show that 50 kHz rat calls are an effective attractant for rats of both sexes under laboratory conditions, while not attracting non-target bank voles. These calls are strong candidates for providing a species-specific lure that may be attractive even in the absence of food bait, but further trials will be needed to assess their efficacy under field conditions.

## Introduction

Globally, many animal species are considered as pests, particularly when they interfere with human activity [1]. This can be in the form of food supply consumption or damage, the spread of disease to humans or livestock, and/or expensive damage to infrastructure [2–4]. In addition, invasive species are classified as pests when they threaten populations of endemic species [4]. Rats, especially brown rats (*Rattus norvegicus*, alternatively called Norway rats) and black rats (*Rattus rattus*), are some of the greatest pests worldwide [3, 4]. They are a major threat to global food security, carry and transmit over 50 zoonotic diseases and pose a significant threat to many endangered species, particularly in island habitats [2, 5–7].

Control of pest species, particularly rats, relies mostly on the use of pesticides [3]. Some of these pesticides are not species-specific and are bio-accumulative, passing up the food chain to affect higher trophic levels [8]. As such, rat poisons are often presented within secure bait boxes to prevent access of pets and children [8]. However, these bait boxes present two main problems. First, it is difficult to attract rats into the unfamiliar enclosed space of a bait box as rats avoid entering them [9]. This avoidance, often termed behavioural resistance, also leads to rats avoiding other control devices that do not require poisons, such as traps [9]. Second, whilst bait boxes prevent access by large non-target animals they do not prevent access by small non-target rodents. Due to this, high numbers of non-target rodents and their predators have been found with residues of rat poisons. This has been linked to poor body condition and fatalities among these non-target species [10–15].

To reduce these problems, we need to reduce the avoidance of control measures by rats and improve their species-specificity. Species-specific attractants targeted at pest species could provide an effective solution. Replication of cues or signals that a pest species normally relies on to locate attractive resources or social opportunities, or to avoid unsuitable conditions, has been identified as having particular potential for exploitation [16, 17]. Indeed, communication signals that play an important role in survival, social coordination and/or reproductive success within a species are likely to be both species-specific and impede the development of resistance [16]. One such signal that has the potential to attract rats is prosocial 50 kHz calls. These calls were discovered by Panksepp and Burgdorf [18] when tickling laboratory rats, and have since been linked to positive emotions (affect) in this species [19]. 50 kHz calls increase during play and sexual encounters, and their emission reduces aggression between conspecifics [20–23]. Rats also appear to use these calls when searching for familiar conspecifics, with calls increasing when cage mates are separated, or when a lone rat is exposed to the scents of other rats from its colony [20, 24]. In open arenas, rats prefer to spend time near to a sound source playing 50 kHz calls. The role of sex in this response has been investigated, but with inconsistent results. [25–30].

Here, we investigate whether 50 kHz rat calls have the potential to act as a potent species-specific attractant and could be a candidate lure that might be exploited for pest control. As the responses of rats to 50 kHz calls have been studied only in open arenas, we test whether calls will attract rats to enter and spend time inside an unfamiliar enclosed space, such as the bait boxes very commonly used in current rat control. Because these pro-social calls are normally used for communication between familiar animals, responses may depend on recognition of the calling rat, which would negate their exploitation as a general attractant. To assess this, we test whether rats are attracted to calls from familiar and unfamiliar individuals. We also examine whether attraction is stronger towards 50 kHz calls from male or female rats, and whether this depends on the respondent’s sex. Although some studies have looked at attraction of both sexes to 50 kHz calls [26, 29, 30], no study has directly compared the response of male and female rats to male and female calls. Lastly, to reduce the exposure of non-target species to rat control measures, attractive lures should be specific to rats. As rats are potential predators of smaller rodents [31, 32], non-target rodents may actively avoid 50 kHz rat calls. As an indication of the response of a typical non-target species affected by rat control [10, 33], we test the response of bank voles (*Myodes glareolus*) to bait boxes emitting 50 kHz rat calls. We find that rat calls (and to a lesser extent, sounds of rat movement) attract other rats to approach and enter empty bait boxes, even when they are completely unfamiliar with the calling rat. By contrast, bank voles are neither attracted nor repelled by 50 kHz rat calls. Our findings under laboratory conditions suggest that 50 kHz calls emitted on separation of familiar cagemates are promising candidate lures for brown rats, although this now needs to be assessed further in field trials.

## Materials and methods

### Ethics statement

All procedures were non-invasive behavioural tests that did not involve pain, suffering, distress or lasting harm and were carried out in accordance with international best practice guidelines [34–37]. The study was approved by the University of Liverpool Animal Welfare Committee. No specific licences were required to carry out the work, as animals were free to approach or avoid the test stimuli. Following use in this study, rat and bank vole subjects were housed for use in further behavioural tests.

### Subjects

Behavioural test subjects were 16 male and 16 female Wistar rats (HsdHan:WIST, Envigo, Bicester, UK) aged 12 to 15 months (Experiments 1 and 2), 8 male and 8 female Wistar rats aged 3 to 4 months (Experiment 3), and 8 male and 8 female bank voles aged 11 to 12 months (Experiment 4). Vocalisation donors were 3 male and 3 female Wistar rats, aged 12 months. Rats were housed in same sex pairs or trios in 56 x 38 x 22 cm cages (RC2R, North Kent Plastics, Coalville, UK), in a room containing both male and female rats. Rats were obtained from Envigo (UK) at 4 to 8 weeks old. Test subject rats were housed in the same room as vocalisation donor rats for Experiments 1 and 2, and in a different room from vocalisation donor rats for Experiment 3. Bank voles (a solitary species) were housed singly in 48 x 15 x 13 cm cages (M3, North Kent Plastics, UK), in a separate room to all rats. Bank voles were first generation, captive bred from wild parents caught in Northwest England. Of the 16 bank vole subjects, 13 (7 females and 6 males) had briefly been exposed to rat odour (but not calls) in a separate behavioural test approximately 6 weeks prior to this study. Neither bank voles nor rats had previous exposure to the test arena or the behavioural test used in these experiments.

All housing and test rooms were maintained at 21 ± 2°C, 55% humidity and 20 air changes per hour. Throughout the test period, rats were housed on a reversed 12:12 hour light-dark cycle with lights off at 0800 h. Bank voles were housed on a reversed 16:8 hour light-dark cycle with lights off at 0900 h. All behavioural experiments were conducted in the dark phase of the light cycle under dim red lights.

All animals were fed 5FL2 EURodent Diet (LabDiet, St Louis, USA) *ad libitum* and had access to *ad libitum* water. All cages had Corn Cob Absorb 10/14 substrate lining the base. Cardboard tubes and paper wool nest material were provided to all animals for enrichment, with 15 x 8 cm plastic tubes also given to rats.

### Composition of playbacks

Playbacks were recorded using a condenser ultrasound microphone CM16/CMPA (Avisoft-Bioacoustics, Glienicke, Germany) connected to an UltraSoundGate 116H (Avisoft-Bioacoustics, Germany). Sound files were recorded and edited to create 15 minute playbacks as follows.

#### Rat playback

50 kHz rat calls were recorded individually from 3 male and 3 female Wistar rats in their own, soiled, home cage after removal of their cage mate, in accordance with Wöhr *et al*. [24]. From each recording, a 30 s section of 50 kHz calls was isolated (S1–S6 Figs). Numbers and types of 50 kHz call for each donor rat were recorded as per Wright *et al*. [38] (S1 Table). No non-50 kHz calls were identified in any of the playbacks. Each playback consisted of this 30 s section of 50 kHz calls on constant loop repeat. Individual playbacks were composed for each of the 6 rats recorded. Selection of playback during testing was randomised.

#### Sound of rat movement playback

We recorded one male Wistar rat during exploration of a home cage, with no vocalisations emitted (S7 Fig). Each playback consisted of 30 s of the sound of rat movements on constant loop repeat.

#### Regularly intermittent white noise playback

We recorded background sound in the room in which rat sounds were recorded (with no animals present) for 15 s. We then generated 15 s of white noise using Avisoft SASLab Pro (Avisoft-Bioacoustics, Germany). White noise volume was matched to the lower end of volume for sound of rat movement playback. The two 15 s sound files were combined to give a 30 s composition (S8 Fig). Each playback consisted of this 30 s composition on constant loop repeat.

The volume of playbacks was adjusted so that they matched the volume initially recorded. This was recorded using a condenser ultrasound microphone CM16/CMPA (Avisoft-Bioacoustics, Germany) connected to an UltraSoundGate 116H (Avisoft-Bioacoustics, Germany). Maximum playback volume was approximately 61 dB for female rat playbacks, 56 dB for male rat playbacks, 48 dB for sound of rat movement playback and 40 dB for white noise playback (measured at 30 cm from sound source).

### Behavioural test of attraction or avoidance

**Fig 1:**
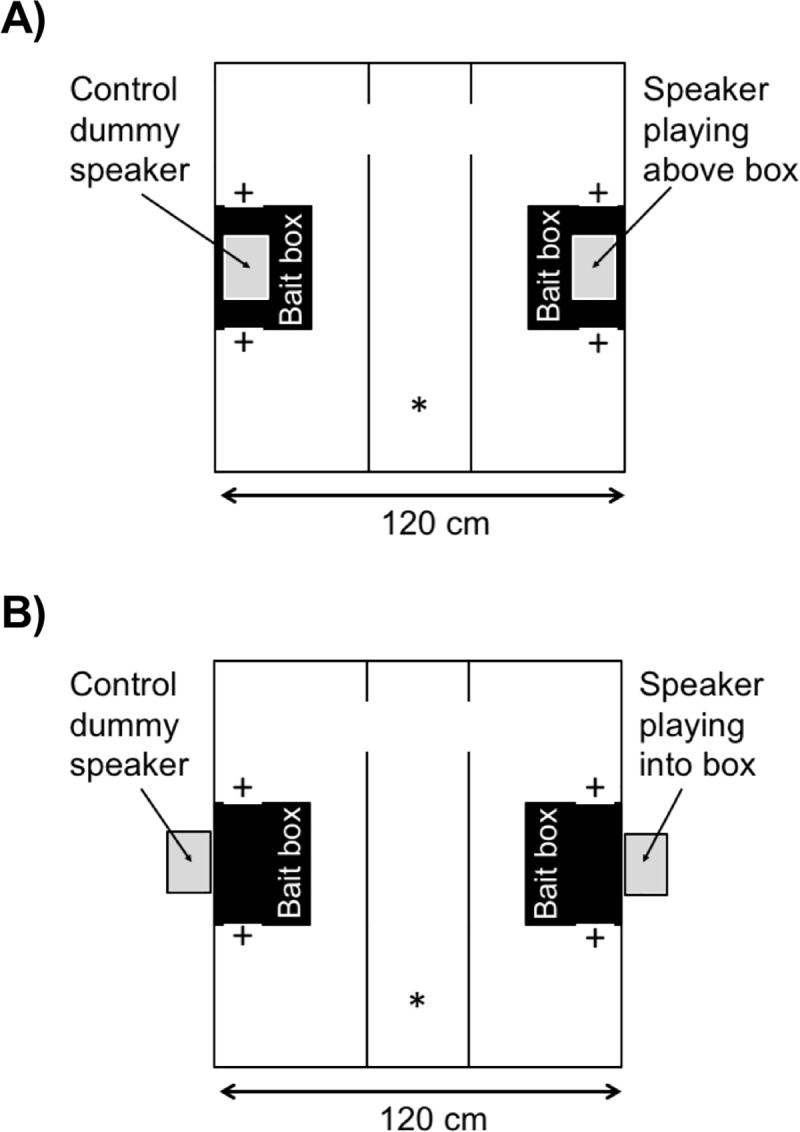
Design of laboratory test arena. A) Speaker placed 50 cm above the bait box. B) Speaker placed outside arena to play through the bait box. * Position of subject placement into arena via handling tunnel; + bait box entrances.

The behavioural test was conducted in a 120 x 120 x 80 cm laminated chipboard arena divided into three sections using polypropylene sheets, with a 45 x 120 cm compartment at each side and a 30 x 120 cm compartment in the middle. The compartments were connected through a 15 x 15 cm gap in the dividing walls towards one end of the arena. A Roguard Extra bait box (28 x 25 x 13 cm, BASF, Cheadle Hulme, UK) was placed against the lateral wall of each side compartment of the arena, such that the dual entrances were closest to the lateral walls (Fig 1A and 1B). A speaker was placed on one side of the arena so that it played either above a bait box (Fig 1A, Experiment 1) or through a bait box (Fig 1B, Experiments 2–4). A matching dummy speaker (empty box of the same dimensions as the speaker) was placed on the other side of the arena. For the speaker (or dummy speaker) to play through a bait box, a 12 cm hole was cut into one side of each bait box, matching up with an 8 cm hole in the arena wall. A wire mesh barrier prevented test subjects from escaping through this hole.

Three experiments used rats as test subjects (n = 16 for each experiment), each tested with three different sound treatments per experiment. The final experiment used bank voles as test subjects (n = 16), each tested with two different sound treatments. An overview of experiments is given in Table 1. The order of test subjects, sound treatments, and side of speaker placement was randomised in a balanced design, with a 6–9 day gap between treatments using the same subject. To minimise stress induced by handling [39, 40], test subjects were moved into and out of the test arena using a clear plastic handling tunnel, capped with wire mesh (33 x 11 cm for rats and 18 x 5 cm for bank voles). Before each treatment, test subjects were familiarised with the arena for 15 minutes with the speaker turned off. Subjects were then confined to a handling tunnel placed outside of the test arena, the speaker was turned on, and subjects were reintroduced into the central compartment of the arena for a 15 minute test period. Subject behaviour was recorded in an adjacent room by remote video monitoring. The arena was cleaned with Teepol Multipurpose detergent (Teepol, Orpington, UK), followed by 70% ethanol, between each subject. Transcription of video recordings was performed blind to sound treatment. We recorded the time spent in each side of the arena, time spent on top of each box and time spent inside each box (S1 Dataset). An animal was recorded as in a location when its whole body (not including the tail) was in that location.

**Table 1:** Overview of experiments.

Experiment number	Test subjects^1^	Test subject age (months)	Familiarity with call donors^2^	Direction of sound	Playbacks
1	Wistar rats	12–15	Familiar	From above box	Female 50 kHz calls Male 50 kHz calls Sound of rat movement
2	Wistar rats	12–15	Familiar	From within box	Female 50 kHz calls Male 50 kHz calls Sound of rat movement
3	Wistar rats	3–4	Unfamiliar	From within box	Female 50 kHz calls Sound of rat movement White noise
4	Bank voles	11–12	Unfamiliar	From within box	Female 50 kHz calls White noise

n = 8 males and 8 females for each experiment.

Familiar: housed in same room, Unfamiliar: housed in separate room.

### Statistical analyses

Bias scores were calculated by subtracting time spent in the silent side of the arena from time spent in the side where sounds were played. Bias scores were calculated for time spent in four locations: total time spent in the arena side, time spent inside boxes, time spent on top of boxes and time spent on the arena floor (in that side of the arena but not interacting with boxes). In addition, bias scores were calculated for the number of entries to bait boxes for Experiments 2 and 3. Time spent in the central compartment of the arena was not included in analyses.

We analysed the effect of sound treatment and test subject sex on bias scores for each of our four locations by two-way repeated measures ANOVA (where data approximated normality) or non-parametric equivalent of a two-way ANOVA (where data was not normally distributed). Where sound treatment had a significant effect, differences between each pair of treatments were assessed using pairwise t-tests with Holm-Bonferroni correction for multiple comparisons (where data approximated normality), or Wilcoxon signed-rank tests with Holm-Bonferroni correction (where data was not normally distributed).

To assess if rats were attracted to, or avoided, sound cues, we analysed bias scores for each of our four locations using one-sample t-tests (where data was normally distributed) or Wilcoxon signed-rank tests (where data was not normally distributed), with Holm-Bonferroni correction where multiple comparisons were made. Where ANOVA results indicated that sound treatment had a significant effect on bias between arena sides, bias observed in each sound treatment was analysed in separate tests. Where results of ANOVA indicated that sound treatment did not have a significant effect on bias between arena sides, all sound treatments were pooled and analysed in one test.

All statistical tests were bidirectional. A p*-*value of less than 0.05 was regarded as significant. Non-parametric equivalents of two-way ANOVAs were carried out using software provided by Barnard *et al*. [41]. All other statistical analyses were carried out using R 3.4.0 [42] with packages ‘ez’ [43], ‘ggplot2’ [44], ‘nortest’ [45], ‘exactRankTests’ [46], ‘extrafont’ [47] and ‘gridExtra’ [48].

## Results

### Experiment 1. Rats are attracted to 50 kHz calls, regardless of sex

To test the sex specificity of 50 kHz rat calls in the attraction of conspecifics, we compared the response of eight male and eight female Wistar rats to three sound treatments: 1) 50 kHz calls from an adult male Wistar rat, 2) 50 kHz calls from an adult female Wistar rat and 3) rat movement sounds, without any calls. Within each treatment, response to the sound playing in one side compartment of the test arena was compared to a silent control in the other side compartment. To test if 50 kHz rat calls attracted rats to move close to a sound source, the speaker or a dummy speaker was placed above an empty bait box present in each side compartment (Fig 1A).

**Fig 2:**
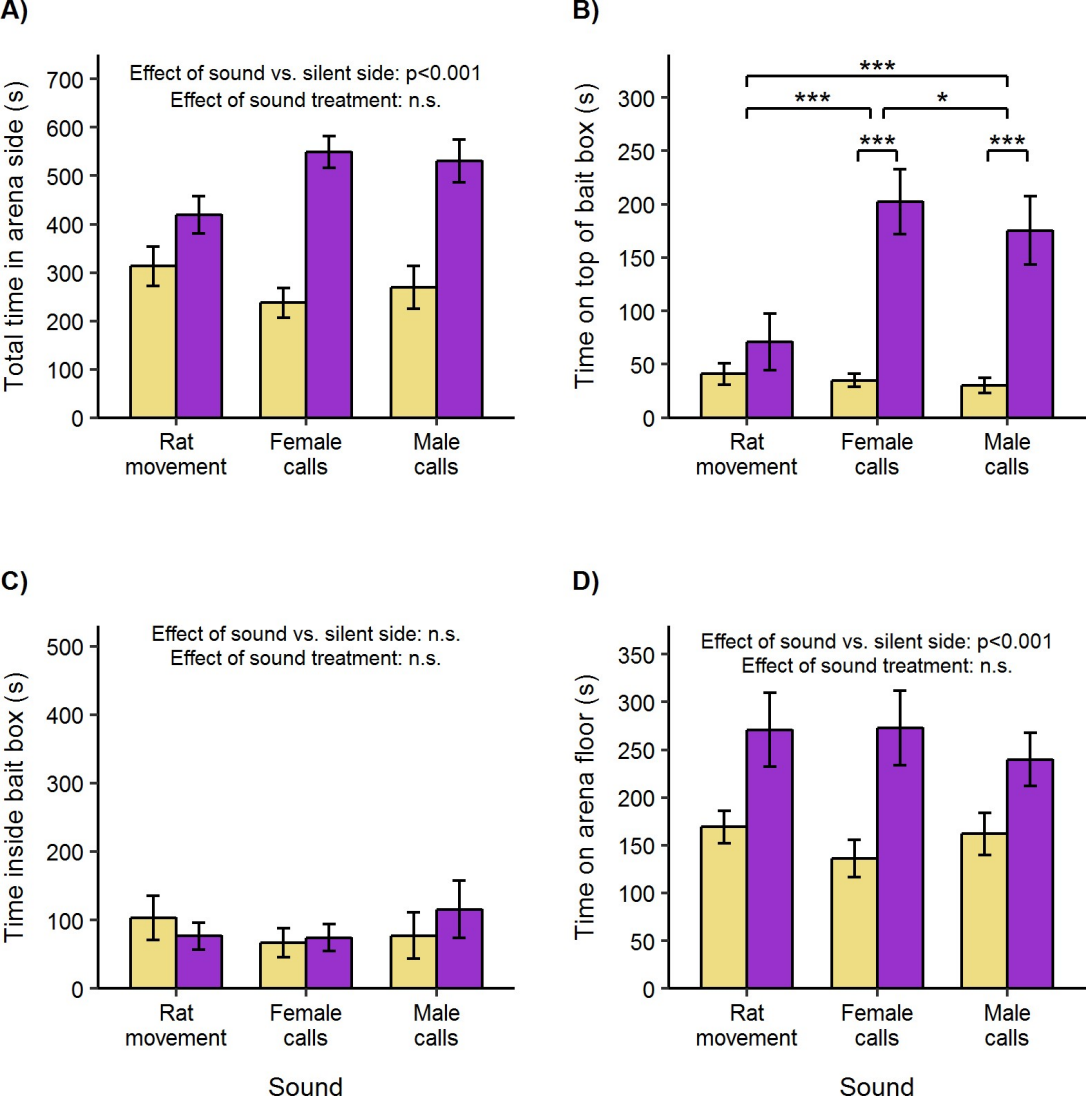
Attraction to rat sounds playing above empty bait boxes (mean ± standard error of mean). Adult male and female Wistar rats were presented with sound playing above an empty bait box (purple bars) versus no sound (yellow bars, control) in opposite side compartments of a test arena. **A)** Total duration in each side compartment of arena. **B)** Duration on top of box. **C)** Duration inside box. **D)** Duration on arena floor (in arena side but not in or on box). Bias between sides was compared between treatments using repeated measures ANOVA followed by pairwise t-tests (where data approximated normality), or non-parametric two-way ANOVA equivalent followed by Wilcoxon signed-rank tests (where data was not normal). Within each treatment, attraction or avoidance was assessed using one-sample t-tests or Wilcoxon signed-rank tests. (*p < 0.05, ***p < 0.001).

Overall, rats were attracted to spend more time in the sound side of the arena (one sample *t*-test, *t*_47_ = 5.05, p < 0.001), regardless of which sound was playing (repeated measures ANOVA, F_2, 28_ = 1.62, p = 0.22) or test subject sex (F_2, 28_ = 0.27, p = 0.76). However, attraction to male and female rat calls appeared to be slightly stronger than attraction to rat movement sounds (Fig 2A).

Looking in more detail at their behaviour, rats spent approximately fivefold more time on top of boxes underneath a speaker playing either male (one sample *t*-test, *t*_15_ = 4.48, p < 0.001) or female rat calls (*t*_15_ = 5.54, p < 0.001) compared to the silent control side. They were not significantly attracted on top of boxes when the speaker played rat movement sounds (*t*_15_ = 1.21, p = 0.24; Fig 2B). Accordingly, in comparison to rat movement sounds played above the bait boxes, rats spent substantially more time close to female rat calls (paired *t-*test, *t*_15_ = 6.02, p < 0.001) and male rat calls (*t*_15_ = 5.23, p < 0.001). They also spent slightly more time on boxes close to female compared to male rat calls (*t*_15_ = 2.13, p = 0.05). Bias in time spent on top of boxes close to the speaker was not influenced by the test subject’s sex (repeated measures ANOVA, F_2, 28_ = 0.16, p = 0.86).

While rats spent more time close to the sound source when rat calls were playing, this did not result in an attraction to spend more time inside boxes (one sample *t*-test, *t*_47_ = 0.29, p = 0.78; Fig 2C). This lack of response was not affected by sound treatment (repeated measures ANOVA, F_2, 28_ = 0.25, p = 0.78) or test subject sex (F_2, 28_ = 0.33, p = 0.72).

Although the strongest response to rat calls playing above the arena was attraction to spend time on top of boxes close to the sound source, rats also spent more time in the vicinity of rat sounds when not on top of or inside boxes (Wilcoxon signed rank test, V = 950, p < 0.001; Fig 2D). In contrast to the close attraction shown specifically towards rat calls (Fig 2B), this more distant attraction was shown equally towards all three types of rat sounds (repeated measures ANOVA, F_2, 28_ = 0.40, p = 0.67), with no difference according to the test subject’s sex (F_2, 28_ = 1.08, p = 0.35). Thus, the sounds of rat movement attracted rats to spend more time in the general vicinity, but only rat calls (from either sex) induced rats to spend time very close to the source of the calls. Behaviour on top of boxes often consisted of stretching upwards, suggesting that they were trying to investigate the source of these calls.

### Experiment 2. Rat sounds attract rats into boxes

To test if sound coming from inside an empty bait box attracts rats to enter the box, the speaker was placed to play inside a box in one side compartment of the arena versus an equivalent silent control in the other side compartment. Eight male and eight female Wistar rats were each tested with three sound treatments: 1) 50 kHz calls from an adult male Wistar rat, 2) 50 kHz calls from an adult female Wistar rat and 3) rat movement sounds, without any calls.

**Fig 3:**
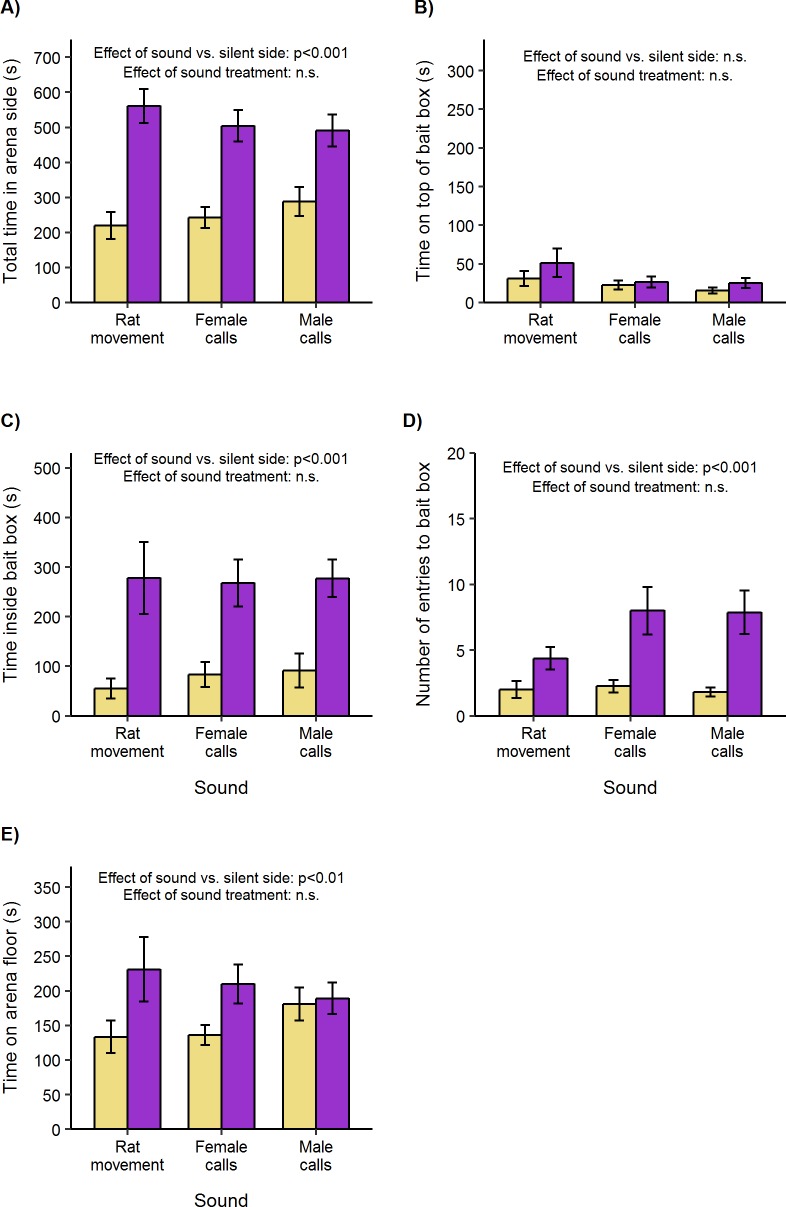
Attraction to rat sounds playing inside empty bait boxes (mean ± standard error of mean). Adult male and female Wistar rats were presented with sound playing through a box (purple bars) versus no sound (yellow bars, control) in opposite sides of a test arena. **A)** Total duration in each side compartment of arena. **B)** Duration on top of box. **C)** Duration inside box. **D)** Number of entries to box. **E)** Duration on arena floor (in arena side but not in or on box). Bias between sides was compared between treatments using repeated measures ANOVA followed by pairwise t-tests (where data approximated normality), or non-parametric two-way ANOVA equivalent followed by Wilcoxon signed-rank tests (where data was not normal). Within each treatment, attraction or avoidance was assessed using one-sample t-tests or Wilcoxon signed-rank tests.

Overall, rats were attracted to spend more time in the sound side of the arena (one sample *t*-test, *t*_47_ = 5.97, p < 0.001; Fig 3A), regardless of which sound was playing (repeated measures ANOVA, F_2, 28_ = 0.91, p = 0.41) or the test subject’s sex (F_2, 28_ = 0.08, p = 0.93). Attraction was mostly due to rats spending more time inside the empty bait boxes (one sample *t*-test, *t*_47_ = 5.12, p < 0.001; Fig 3C), with no difference in response to male or female calls or to the sound of rat movement (repeated measures ANOVA, F_2, 28_ = 0.13, p = 0.88). The strength of attraction did not differ according to the test subject’s sex (repeated measures ANOVA, F_2, 28_ = 0.25, p = 0.78).

In addition to spending more total time inside boxes, rat sounds attracted rats to enter boxes more frequently (Wilcoxon signed rank test of sound versus silent box, V = 758.5, p < 0.001), with no difference between the three sound treatments (non-parametric two-way ANOVA equivalent, H_2, 28_ = 3.8, p = 0.15; Fig 3D). However, female rats showed a greater positive bias in entering boxes where sounds were playing than male rats for all three treatment sounds (median bias ± IQR: female, 6 ± 6.5; male, 2 ± 4.25; non-parametric two-way ANOVA equivalent, H_1, 14_ = 5.59, p = 0.02).

When sounds were played from inside the empty bait boxes, rats spent no more time on top of the box in the sound side of arena compared to the silent side of the arena (Wilcoxon signed rank test, V = 437, p = 0.10; Fig 3B). This lack of response was not influenced by sound treatment (non-parametric two-way ANOVA equivalent, H_2, 28_ = 0.78, p = 0.68) or test subject sex (H_2, 28_ = 2.44, p = 0.30). However, rats were attracted to spend slightly more time in the vicinity of rat sounds when not inside or on boxes (Wilcoxon signed rank test, V = 194, p = 0.002; Fig 3E), a bias that did not differ according to the type of sound (non-parametric two-way ANOVA equivalent, H_2, 28_ = 0.15, p = 0.93) or sex of test subject (non-parametric two-way ANOVA equivalent, H_2, 28_ = 1.01, p = 0.60).

### Experiment 3. Unfamiliar female 50 kHz calls attract rats into boxes

In both Experiment 1 and Experiment 2, test subjects were housed in the same room as the rats that provided the sound cues tested. As familiarity with calls from the same donors might be responsible for the attraction we observed, in this experiment we tested rats that had no prior exposure to the individuals that provided the sound stimuli. Further, the previous experiments revealed attraction to both 50 kHz rat calls and the sounds of a rat moving around without calling. To confirm that rats are attracted to the sounds of rats specifically and not just to the presence of any sound cue, we also tested the response of rats to regularly intermittent white noise. Eight male and eight female Wistar rats were each tested with three sound treatments played through an empty bait box: 1) 50 kHz calls from an adult female Wistar rat, 2) rat movement sounds, without any calls and 3) regularly intermittent white noise. Female rat calls were chosen, as these were slightly more attractive to rats of both sexes than male rat calls in Experiment 1.

**Fig 4:**
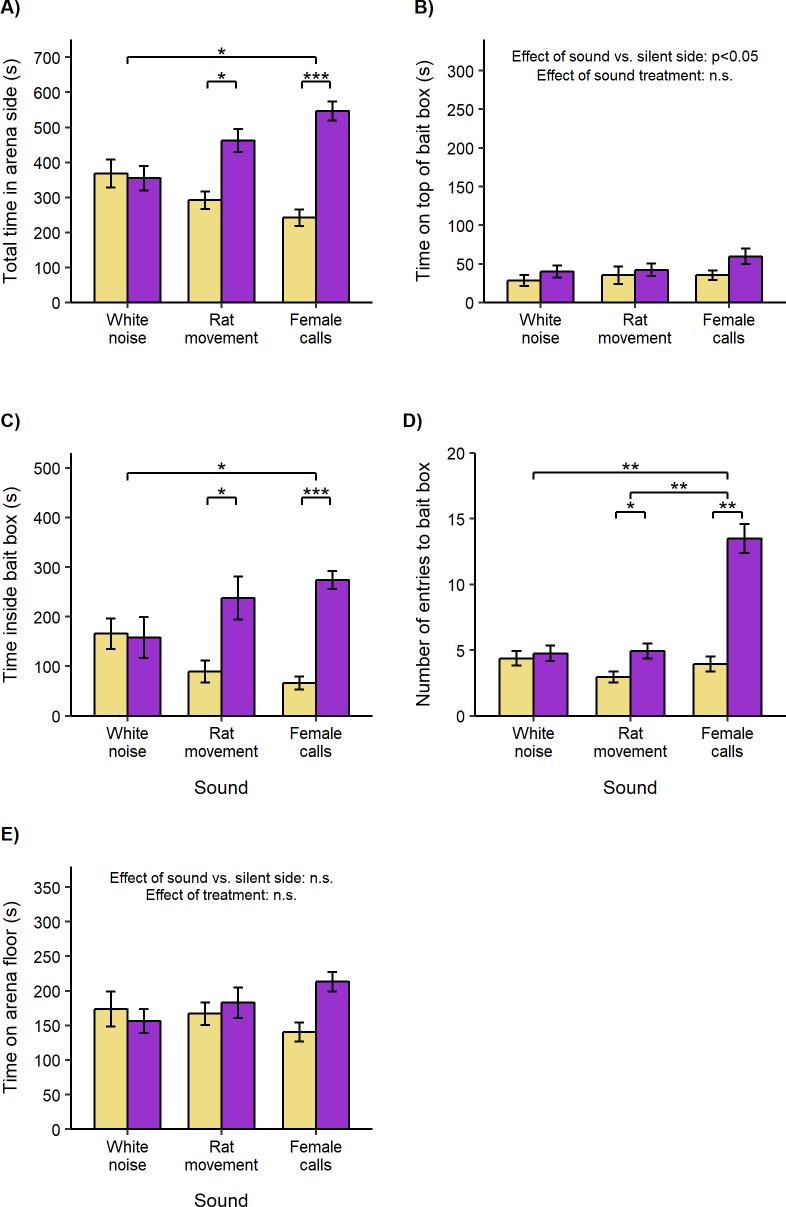
Attraction to rat sounds from unfamiliar donors playing inside empty bait boxes compared to white noise (mean ± standard error of mean). Adult male and female Wistar rats were presented with sound playing through a box (purple bars) versus no sound (yellow bars, control) in opposite sides of a test arena. **A)** Total duration in each side compartment of arena. **B)** Duration on top of box. **C)** Duration inside box. **D)** Number of entries to box. **E)** Duration on arena floor (in arena side but not in or on box). Bias between sides was compared between treatments using repeated measures ANOVA followed by pairwise t-tests (where data approximated normality), or non-parametric two-way ANOVA equivalent followed by Wilcoxon signed-rank tests (where data was not normal). Within each treatment, attraction or avoidance was assessed using one-sample t-tests or Wilcoxon signed-rank tests. (*p < 0.05, **p < 0.01, ***p < 0.001).

Overall, bias in the total time spent in the two side compartments of the arena differed between sound treatments (repeated measures ANOVA, F_2, 28_ = 5.74, p = 0.008) but did not depend on the test subject’s sex (repeated measures ANOVA, F_2, 28_ = 0.22, p = 0.81). Rats were attracted by female rat calls (one sample *t*-test, *t*_15_ = 6.10, p < 0.001), and by rat movement sounds (*t*_15_ = 3.11, p = 0.014), but not by white noise (*t*_15_ = -0.18, p = 0.86; Fig 4A). We confirmed that attraction to the side with female rat calls was significantly stronger than to white noise (paired *t-*test, *t*_15_ = 3.12, p = 0.02). Attraction to the side playing rat movement sounds was intermediate and did not differ significantly from either female calls (*t*_15_ = 1.77, p = 0.15) or white noise (*t*_15_ = 1.93, p = 0.15).

Attraction to spend more time near rat sounds was explained by time spent inside boxes close to the sound source and did not depend on the test subject’s sex (repeated measures ANOVA, F_2, 28_ = 0.07, p = 0.93). Rats spent more time inside bait boxes playing female rat calls (one sample *t*-test, *t*_15_ = 7.84, p < 0.001) or rat movement sounds (*t*_15_ = 2.94, p = 0.02), but not inside those playing white noise (*t*_15_ = -0.13, p = 0.90; Fig 4C). Again, we confirmed that attraction to female rat calls was significantly stronger than to white noise (paired *t-*test, *t*_15_ = 3.19, p = 0.02), but rat movement sounds stimulated an intermediate response, not differing significantly from either female rat calls (*t*_15_ = 1.09, p = 0.29) or white noise (*t*_15_ = 1.76, p = 0.20).

As well as attracting rats to spend more time inside boxes, female rat calls attracted rats to enter boxes more frequently (Wilcoxon signed rank test, V = 136, p = 0.001; Fig 4D). No bias in entry was observed when white noise was played (V = 65, p = 0.44), while the bias in entry stimulated by female rat calls was significantly stronger that induced by the white noise control (V = 1, p = 0.002). Although rat movement sounds also stimulated a small positive entry bias (V = 91.5, p = 0.03), this was much less than entries stimulated by female rat calls (V = 1, p = 0.002) and did not differ significantly from white noise (V = 85.5, p = 0.15). Interestingly, male rats entered boxes where sounds were playing slightly more frequently than females across all sound treatments (median bias ± IQR: male, 3.5 ± 9; female, 2 ± 5; non-parametric two-way ANOVA equivalent, H_1, 14_ = 25.32, p < 0.001).

There was a small increase in time spent on top of boxes in the side of the arena where sounds were played compared to the silent control side (one sample *t*-test, *t*_47_ = 2.49, p < 0.016; Fig 4B), which was not influenced by sound treatment type (repeated measures ANOVA, F_2, 28_ = 0.68, p = 0.52) or test subject sex (F_2, 28_ = 0.11, p = 0.90). However, sound treatment did not influence time in the vicinity but away from the boxes (Fig 4E).

### Experiment 4. Bank voles are neither attracted to, nor repelled by, 50 kHz rat calls

As a first step towards establishing the species specificity of attraction to 50 kHz rat calls, we tested the response of heterospecific bank voles to rat calls played through an empty bait box versus a silent control box. Eight male and eight female bank voles were each tested with two sound treatments: 1) 50 kHz calls from an adult female Wistar rat and 2) regularly intermittent white noise.

**Fig 5:**
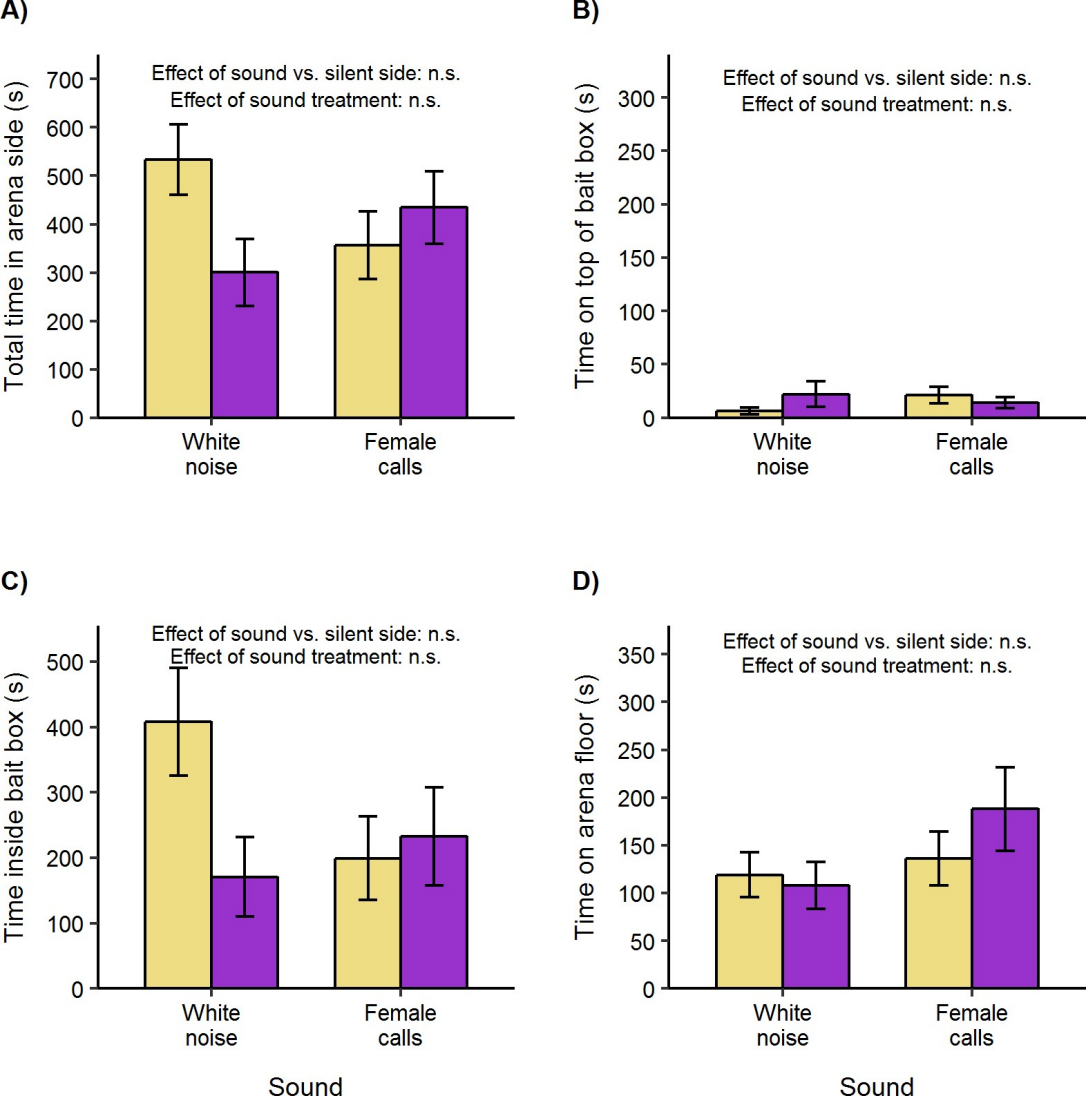
Bank vole response to female rat calls or to white noise playing inside empty bait boxes (mean ± standard error of mean). Adult male and female bank voles were presented with sound playing through a box (purple bars) versus no sound (yellow bars, control) in opposite sides of a test arena. **A)** Total duration in each side compartment of arena. **B)** Duration on top of box. **C)** Duration inside box. **D)** Duration on arena floor (in arena side but not in or on box). Bias between sides was compared between treatments using repeated measures ANOVA followed by pairwise t-tests (where data approximated normality), or non-parametric two-way ANOVA equivalent followed by Wilcoxon signed-rank tests (where data was not normal). Within each treatment, attraction or avoidance was assessed using one-sample t-tests or Wilcoxon signed-rank tests.

Overall, bank voles showed no bias in total time spent in the two side compartments of the test arena (one sample *t*-test, *t*_31_ = -0.75, p = 0.46; Fig 5A), with no difference in response to female rat calls or white noise (repeated measures ANOVA, sound F_1, 14_ = 1.96, p = 0.18). Subject sex did not influence this lack of response (F_1, 14_ = 0.05, p = 0.83; interaction between sound and sex, F_1, 14_ = 0.08, p = 0.78). There were also no differences in the more specific behaviours of time inside boxes (Fig 5C), time on top of boxes (Fig 5B) or time in the vicinity away from boxes (Fig 5D).

## Discussion

We found that rats of both sexes were attracted to 50 kHz calls from either sex and to sounds of a rat moving around without vocalising. However, 50 kHz rat calls stimulated more consistent attraction than the sounds of rat movement alone. Whilst both types of rat sounds attracted rats to spend time in the general vicinity of a sound source, only 50 kHz calls attracted rats to spend time on top of boxes when sounds were played above the boxes. Notably, when listening rats were unfamiliar with sound donor rats, attraction to female 50 kHz calls was very strong while rat movement sounds stimulated only a weak attraction that was not significantly greater than response to white noise. 50 kHz calls playing inside bait boxes stimulated both a greater number of entries and much more time spent inside bait boxes, despite the absence of any food baits in our tests. We found no sex specificity in the responses of rats to 50 kHz calls or rat movement sounds, although there was some evidence that 50 kHz calls from females were slightly more attractive than those from males.

The attraction of rats to 50 kHz rat calls in this study is consistent with the findings of several previous studies that looked at attraction, though not in the context of using rat calls as a potential lure. In a series of experiments using a radial arm maze, male 50 kHz calls attracted adults of both sexes and juvenile rats (only males tested) into arms of the maze [25, 26, 28, 49]. In addition, Sadananda *et al*. [27] showed male attraction to male 50 kHz calls in a compartmentalised arena similar to that used in the current study but without empty bait boxes. All of these studies used laboratory rats of a Wistar strain, recorded 50 kHz vocalisations in a manner similar to that employed here and used sounds of rat movement as a control. In the current study, we found that rats were attracted into boxes by both 50 kHz calls (which included rat movement sounds in the background) and rat movement sounds alone. Thus, we cannot conclude that attraction in our experiments was attributable to 50 kHz rat calls alone, although previous studies did not find attraction to background movement sounds. However, we found that attraction to rat movement sounds was reduced in Experiment 3, when subjects were not familiar with sound donors, whereas in Experiment 2 subjects were kept in the same room as sound donors. The reduced strength of attraction to rat movement sounds could be because of reduced familiarity with the sound donor, or because test subjects were 12–15 months old in Experiment 2 but only 3–4 months old in Experiment 3. Older rats housed over a prolonged period in laboratory cages might learn a stronger association between the incidental sounds of rats moving around a cage and other visual, auditory or contact cues that indicate the presence of another rat. Previous studies that used rat movement sounds as a control sound in radial arm maze experiments [26] recorded these in the same manner as our current study and found no attraction among rats tested at 3–4 weeks or 3–4 months of age. The context of presentation may also affect attraction to the incidental sounds of rat movement, which are not a fixed cue produced by rats but influenced by the background environment. In an open environment, such as in Experiment 1 or the arenas used by other researchers, rats may be attracted only to cues that are specific to another rat, particularly when they had to climb up onto boxes to approach the source. However, when sounds are played within an enclosed space, as in Experiments 2 and 3, rats may be attracted by any sound that suggests the presence of another animal. By contrast, rats were consistently attracted to 50 kHz calls in all experiments, spending substantially more time in close vicinity to the source of these calls, even when this required entering an enclosed space or climbing onto a box where they otherwise spent little time.

We found no sex specificity in the attraction of rats to 50 kHz rat calls. In contrast to our findings, Snoeren and Ågmo [29] observed that female 50 kHz calls were not attractive to male rats. The experimental design used by Snoeren and Ågmo [29] differed from our design in two important aspects. Snoeren and Ågmo [29] used an open single compartment arena, of similar size to one side of the test arena used in our current experiment, with auditory stimuli played through a mesh opening in the arena wall. It is possible that spatial preference for the sound source is less evident when rats are tested in a smaller open area. However, in a follow-up study using the same single compartment arena design, Snoeren and Ågmo [30] found that male 50 kHz calls produced during sexual encounters were attractive to female rats. It is more likely that the difference is due to the calls used as stimuli in these experiments. Snoeren and Ågmo [29] recorded 50 kHz calls when a female rat was sniffing a male rat, in an arena where the female had previously copulated with a male. This contrasts with the 50 kHz calls we recorded on separation of same-sex cage mates. Rat calls in the 50 kHz range have been categorised into 14 different subtypes, with different subtypes assigned to sexual encounters versus cage exploration [38]. It is likely that different call subtypes produced in such different social contexts will elicit different behavioural responses. This implies that the 50 kHz calls that we recorded during separation of same-sex cage mates are likely to provide a more effective attractant for control purposes than calls recorded during sexual encounters, as the calls that we used were strongly attractive to both sexes.

Bank voles were neither attracted to, nor repelled by 50 kHz rat calls. This is the first study to examine the effects of 50 kHz rat calls on heterospecifics. As rats are potential predators of small rodents [31, 32], we predicted that bank voles might avoid ultrasonic rat calls. The observed lack of response is unlikely to be because bank voles were unable to hear calls in this range as they themselves emit calls between 20 and 50 kHz [50, 51]. However, bank voles may learn to avoid cues from dangerous heterospecifics rather than show innate avoidance. The bank voles we used were first generation, captive bred animals that had no experience of rat vocalisations prior to this study. It is also possible that bank voles use other sensory modalities, such as odours either alone or in combination with other cues, as a reliable alert to the presence of rats, rather than using sound cues alone. Our findings suggest that 50 kHz rat calls are unlikely to provide a strong repellent that would keep bank voles away from palatable rat baits. However, using 50 kHz calls as a lure for rats is also unlikely to exacerbate the exposure of bank voles to control measures targeted at rats. The responses of other non-target rodent species will need to be tested to establish whether other species show a similar lack of response. As most non-target rodents are smaller than rats and vulnerable to attack, it seems unlikely that many would be attracted by rat calls.

The consistent attraction of unfamiliar rats of either sex to the 50 kHz calls emitted when rats are separated from same-sex cagemates, including attraction to enter and spend time inside bait boxes in the absence of attractive food, suggests that these calls could have potential application as an attractant lure in the context of rat pest control. However, further research will be needed to establish how far these findings translate to pest control settings. Our study involved laboratory rats tested in laboratory arenas. Little if anything is yet known about the production and use of 50 kHz calls among wild rats. A comparison of ultrasonic calls produced by laboratory-bred versus free-living California mice (*Peromyscus californicus)* revealed that the free-living mice produced higher frequency and more variable calls [52]. Calls most similar to those produced by unstressed wild rats are likely to be the most effective lure. The slightly stronger attraction to 50 kHz calls from females in our laboratory study suggests that female calls should be trialled as a priority in field experiments. It is also possible that wild rats may be most effectively attracted using a combination of cues. A recent field study showed that synthesized ultrasonic calls imitating those of infant rats increased the number of female rats captured in trap boxes also baited with food and female rat soiled bedding [53]. Removal of either the infant calls or the odour cue significantly reduced the number of rats captured. However, as infant calls were not tested alone, it is not known whether infant calls would attract rats into enclosed control points in the absence of bait [53]. 50 kHz prosocial calls may provide an advantage over infant calls in that rats of both sexes readily emit these calls and in turn are attracted to enter boxes playing these calls, providing a natural attractant that is much less likely to depend on the sex and breeding status of respondents. An effective rat lure based on these communication signals would be particularly beneficial in situations where standard control measures using food baits are not successful in eradicating all individuals, typically because some rats will not enter bait or trap boxes, or to check that no individuals remain. In habitats where alternative food is readily available, food baits alone may be relatively ineffective at attracting rats into bait stations [54]. Lures that can effectively entice rats into traps could have the additional benefit of reducing reliance on anticoagulant rodenticides, currently the predominant method used for rat control because rats are typically extremely wary of approaching traps [3, 9, 55]. Even if rat calls do not repel non-target rodents such as bank voles from attractive rodenticide baits, an ability to control of rats more efficiently, combined with a reduced reliance on highly toxic and persistent second generation anticoagulant rodenticides, will help to reduce the environmental impact of rat control.

In conclusion, our study has shown that 50 kHz rat calls, combined with rat movement sounds, are attractive to both male and female rats under laboratory conditions. These calls draw rats to approach, and to enter and spend time in empty bait boxes, with female calls being slightly more attractive than male calls. In addition, we found that bank voles appear to be neither attracted nor repelled by these calls. This suggests that the 50 kHz calls that rats emit on separation from familiar companions are promising candidates for providing an effective species-specific lure that could help to improve the efficacy of rat control and reduce its environmental impact. Further trials are now required to test efficacy under field conditions.

## Supporting information

S1 Fig Playback recording for male rat 14448. (TIF)

S2 Fig Playback recording for male rat 14449. (TIF)

S3 Fig Playback recording for male rat 14456. (TIF)

S4 Fig Playback recording for female rat 14465. (TIF)

S5 Fig Playback recording for female rat 14466. (TIF)

S6 Fig Playback recording for female rat 14467. (TIF)

S7 Fig Sound of rat movement playback recording. (TIF)

S8 Fig Regularly intermittent white noise playback. (TIF)

S1 Table Numbers and types of 50 kHz rat calls for each donor rat. (XLSX)

S1 Dataset Full behavioural test results for each experiment. (XLSX)
